# Ozone-Oxidation of Glucose to Formic Acid over Polyoxmetalates

**DOI:** 10.3390/molecules31030467

**Published:** 2026-01-29

**Authors:** Xia Yu, Qiwen Wang, Haiyan Li, Tong Liu, Mengxue Xiu, Baiji Xue, Linghe He

**Affiliations:** 1 Pharmaceutical Research and Development Center, Baicheng Medical College, Baicheng 137000, China; yux123@nenu.edu.cn (X.Y.); 18904362773@163.com (H.L.); 13351525182@163.com (T.L.); xmx@bcmc.edu.cn (M.X.); xuebj333627@163.com (B.X.); 2 College of Resources and Environment, Jilin Agricultural University, Changchun 130118, China

**Keywords:** polyoxometalates, ozone (O_3_), glucose, oxidation, formic acid

## Abstract

The efficient oxidation of glucose to formic acid (FA) has emerged as a sustainable method for biomass utilization. Herein, we developed a new approach to fulfill oxidation of glucose to formic acid using a polyoxometalate (POM) K_10_SiW_9_Mn_3_^II^O_37_/O_3_ system, and its high efficiency was presented with 79.3% yield of FA at 82.1% conversion at room temperature for 3 h. As evidenced by experiments, the components in Mn-POMs significantly influenced glucose conversion due to their effect on generating reactive oxygen species (ROSs) from O_3_, which was essential for FA production.

## 1. Introduction

Polyoxometalates (POMs) are classic metal–oxide clusters, which show great potentials in catalysis [1,2,3], especially in biomass utilization [4]. In recent years, many efforts have been made in the conversion of biomass into value-added fine chemicals to replace petroleum-based chemicals in industry [5]. In particular, the production of formic acid (FA) from monosaccharides or polysaccharides over POMs has attracted significant attention due to its wide applications in chemicals, pharmaceutical and textile industries [6,7,8]. H_5_PV_2_Mo_10_O_40_ was the first catalyst for the oxidation of biomass to FA using O_2_ as an oxidant [9]. Subsequently, H_8_PMo_7_V_5_O_40_ was used as a homogeneous catalyst to produce FA from glucose with 2 MPa of O_2_, wherein an 85% yield was achieved using a long-chain primary alcohol as an extractant [10]. The use of an extracting agent not only isolated the produced FA, but also limited the decrease in pH, which favored the enhancement of FA selectivity. However, the main challenge in the field lay in increasing FA selectivity and preventing over oxidation to CO_2_ and H_2_O [11,12]. Furthermore, Wu et al. developed a new looping oxidant process using H_8_PMo_7_V_5_O_40_ as an oxygen carrier [13,14], wherein a 95.4% yield of FA was achieved in the oxidation of glucose. This success was attributed to the separated processes for glucose oxidation and regeneration of reduced H_8_PMo_7_V_5_O_40_. To date, there have been few reports on the development of more POMs for the oxidation of glucose to FA (Table S1) [15,16,17,18,19,20,21,22,23,24,25,26]. Transition metal (TM)-substituted POMs could provide a greater chance of developing new oxidative catalysts suitable for production of FA from mono- or polysaccharides, using oxygen or other oxidants.

To date, catalytic ozonation (CO) has attracted significant attention in pollutant abatement [27], whereas less attention has been paid to organic transformation [28,29,30]. By now, some metal catalysts including Mn, Fe oxides or complexes had been developed for ozonation of organic pollutants. To the best of our knowledge, there are currently no reports on ozonation based on POMs. Due to the incorporation effect of TM on vacant POMs, Mn-substituted POMs including K_x_[SiW_12−n_Mn_n_^m^O_(40−n)_] (abbreviated as SiW_12−n_Mn_n_^m^, n = 1–3, m = II–IV) [31,32,33,34,35,36,37] could be easily synthesized to control the compositions of Mn in POMs. As such, SiW_12−n_Mn_n_^m^ might provide some new concepts for oxidation in the presence of ozone.

Herein, we developed POMs containing Mn including K_x_[SiW_12−n_Mn_n_^m^O_(40−n)_], which showed activity in glucose oxidation to FA in the presence of O_3_. An 82.1% conversion and 79.3% yield were achieved at room temperature for 3 h in water. And the mechanism study demonstrated that reactive oxygen species (ROSs) were generated during ozonation, which contributed to high conversion of monosaccharides as well as cellulose. These results have great potential in FA production, as well as the application of O_3_ in the industry for POMs. This work also provides valuable insights into the application of POMs in catalytic ozonation of glucose or other saccharides into FA.

## 2. Results and Discussion

**Figure 1:**
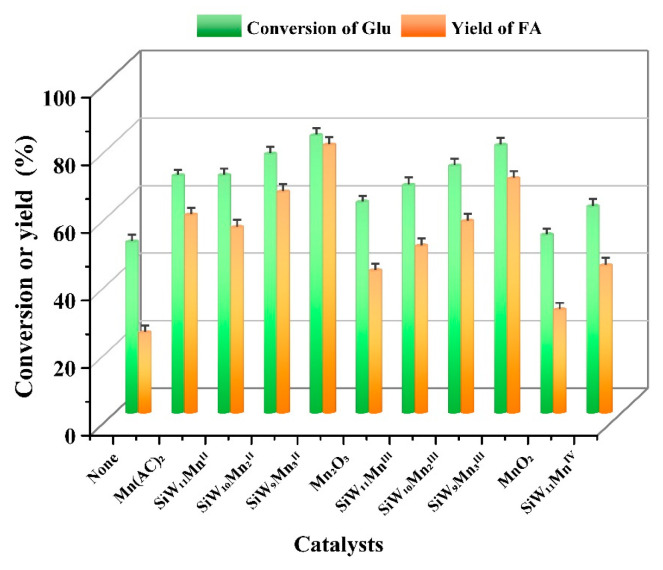
Catalytic activity of various catalysts. Reaction conditions: 100 mg glucose, 10 mg catalyst, 10 mL water, O_3_ flow rate of 30 mL/min for 3 h at room temperature.

Firstly, we screened the catalytic activity of various Mn-POMs in ozonation of glucose under the following reaction conditions: 100 mg of glucose, 10 mg of catalyst, 10 mL of water, an O_3_ flow rate of 30 mL/min, for 3 h at room temperature (Figure 1). Without any catalyst, O_3_ oxidized glucose to 50.6% conversion, but only 46.8% selectivity to FA. This indicates the high oxidative ability of O_3_ for glucose oxidation, but with lower selectivity. It was observed that Mn-POMs exhibited catalytic activity in the ozonation of glucose in the order of SiW_12_ (51.3%) < SiW_9_ (54.4%) < SiW_11_Mn^II^ (69.3%) < SiW_10_Mn_2_^II^ (76.6%) < SiW_9_Mn_3_^II^ (82.1%). This indicated that Mn-POMs could catalyze O_3_ to oxidize glucose at room temperature, and their activity depended on the Mn substitution of W. In contrast, silicotungstate SiW_12_ did not show any activity in the ozonation of glucose, indicating the essential role of Mn in POMs for this reaction. Furthermore, the activity of Mn-POMs relied on the account of Mn components in POMs, with SiW_9_Mn_3_^II^ showing the highest activity among all Mn-POMs. Compared with Mn(OAc)_2_, SiW_9_Mn_3_^II^ presented enhanced activity, which was attributed to the effect of polyanion. To investigate this, we checked the activity of SiW_12−n_Mn_n_^III^ with a higher valence state in ozonation of glucose. It was observed that Mn^III^-POM showed relatively lower activity than Mn^II^-POM did. This implied that Mn^II^-POM was first oxidized by O_3_ to Mn^III^-POM, and the oxidized form then reacted with glucose to cleave C-C or C-O bonds. During this process, Mn^III^-POM was reduced back to the original Mn^II^-POM to complete the catalytic cycle. The lower activity of Mn^III^-POM was likely because the ozonation of Mn^III^-POMs to Mn^IV^-POMs might be more difficult compared to the oxidation between Mn^II^-POMs to Mn^IV^-POMs, which induced the lower activity for Mn^III^-POMs compared to Mn^II^-POMs [38]. Meanwhile, the oxidation of Mn^IV^-POMs to high-valence Mn^V^ became more difficult [39].

For the undecatungstosilicate, Mn could be coordinated with mono-lacunary SiW_11_ to form SiW_11_Mn^II^, which was oxidized by O_3_ or K_2_S_2_O_8_ to SiW_11_Mn^III^ and SiW_11_Mn^IV^. The catalytic activity followed the order of 69.3%(SiW_11_Mn^II^) > 67.4% (SiW_11_Mn^III^) > 61.1% (SiW_11_Mn^IV^) in ozonation of glucose. Based on these results, we assumed the mechanism was as follows: (1) O_3_ reacted with SiW_11_Mn^II^ to [SiW_11_Mn^III^-O_3_·] via oxidation–reduction [40]; (2) release one O_2_ molecule to [SiW_11_Mn^III^-O·]; (3) coupled with a H^+^ to [SiW_11_Mn^III^-·OH] [41]; (4) finally ·OH was removed from [SiW_11_Mn^III^-·OH] to SiW_11_Mn^III^, and ·OH oxidized glucose; and (5) SiW_11_Mn^III^ participated the oxidation of glucose, while SiW_11_Mn^III^ accepted 1e^−^ to fresh SiW_11_Mn^II^. The relevant equations are listed as follows:

SiW_11_Mn^II^ + O_3_ → SiW_11_Mn^III^-O_3_· (1)

SiW_11_Mn^III^-O_3_· → SiW_11_Mn^III^-O· + O_2_ (2)

SiW_11_Mn^III^-O· + H^+^ → SiW_11_Mn^III^-·OH (3)

SiW_11_Mn^III^-·OH → SiW_11_Mn^III^ + ·OH (4)

glucose + ·OH + SiW_11_Mn^III^ → FA + SiW_11_Mn^II^ + H_2_O (5)

**Figure 2:**
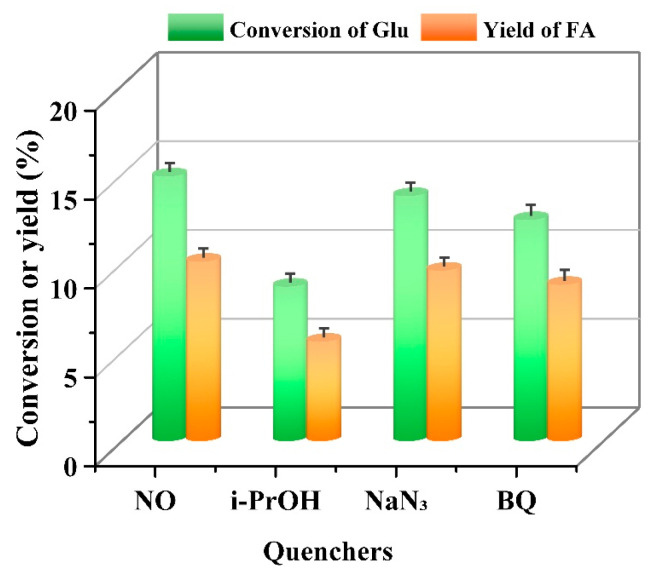
Quenching experiments for SiW_9_Mn_n_^m^ (valence of m, n = 2, 3) in the ozonation of glucose. Reaction conditions: 100 mg glucose, 10 mg catalyst, 10 mL water, O_3_ flow rate of 30 mL/min for 0.5 h at room temperature.

Based on the above results, SiW_11_Mn^II^ appears to be the most active among SiW_11_Mn^II^, SiW_11_Mn^III^ and SiW_11_Mn^IV^, whereas SiW_11_Mn^IV^ was difficult to oxidize to high-valence Mn in POMs using O_3_. This hypothesis was further verified by a reactive oxygen species (ROS) quenching test. As shown in Figure 2, isopropanol (i-PrOH), sodium azide (NaN_3_) and p-benzoquinone (BQ) were employed as scavengers to trap ·OH, ^1^O_2_ and ·O_2_^−^, respectively. It was observed that the conversion of glucose was significantly inhibited by addition of i-PrOH, whereas NaN_3_ and BQ had a negligible influence on catalytic activity of SiW_9_Mn_3_^II^. This indicated that ·OH was the primary ROS during the ozonation process in the presence of Mn-POMs. Minor amounts of ·O_2_^−^ and ^1^O_2_ also contributed to the reaction involving O_3_ and ·OH [30]:

O_3_ + ·OH → HO_2_· + ·O_2_^−^ (6)

And ·O_2_^−^ dismutated into ^1^O_2_ [42]:

2·O_2_^−^ → ^1^O_2_ + O_2_^2−^ + ^3^O_2_ (7)

**Figure 3:**
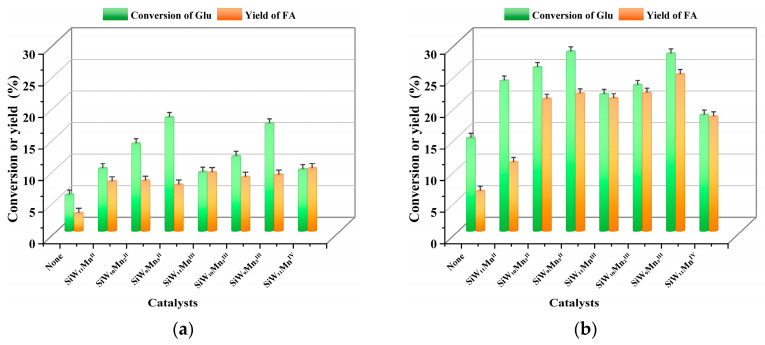
Influence of i-PrOH in the ozonation activity of Mn-POMs. Reaction conditions: 100 mg of glucose, 10 mg of catalyst, 1 mg of i-PrOH, 10 mL H_2_O, room temperature, O_3_ flow rate of 10 mL/min, for (**a**) 0.5 h and (**b**) 1.0 h.

The generation of ·OH was dependent on the species of Mn POMs (Figure 3). Notably, SiW_9_Mn_3_^II^ exhibited the highest activity in generating ·OH, which is consistent with its superior performance in glucose conversion. Furthermore, ·OH radical production correlated with the amount and valence of Mn, following the trend: SiW_9_Mn_3_^II^ > SiW_10_Mn_2_^II^ > SiW_11_Mn^II^ and SiW_9_Mn_3_^II^ > SiW_9_Mn_3_^III^. Consequently, SiW_9_Mn_3_^II^ effectively induced the decomposition of O_3_ to generate ·OH, along with ·O_2_^−^ and ^1^O_2_, confirming that ·OH played a pivotal role in glucose oxidation.

**Figure 4:**
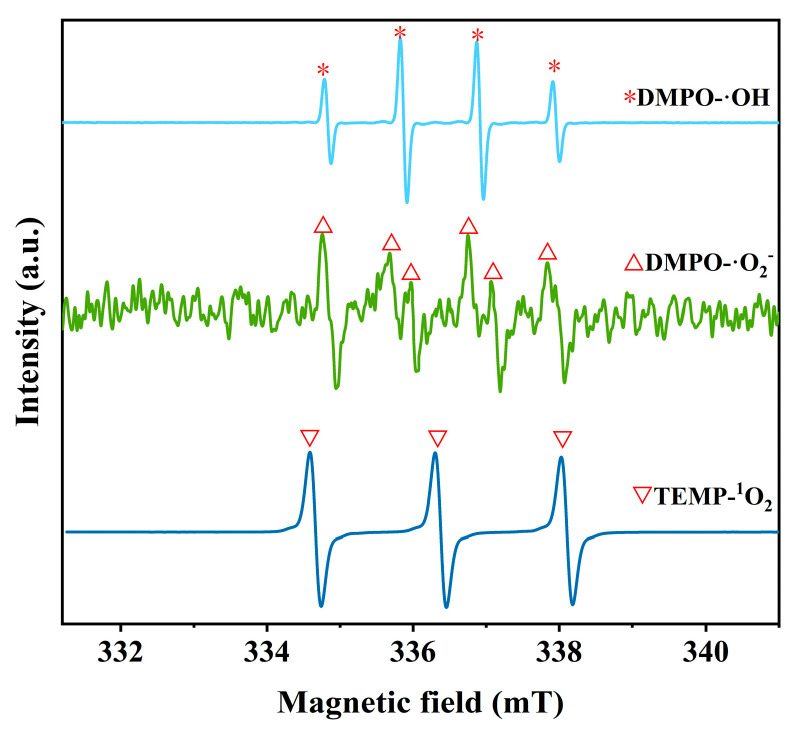
EPR spectra of DMPO-·OH (*) in water, DMPO-·O_2_^−^ (△) in methanol and TEMP-^1^O_2_ (▽) in water. Reaction condition: 10 mg SiW_9_Mn_3_^II^, 50 mM DMPO or TEMP, 10 mg/L O_3_ and room temperature.

Furthermore, Electron Paramagnetic Resonance (EPR) spectroscopy was employed to confirm the production of ROSs in the presence of O_3_ and SiW_9_Mn_3_^II^. 5,5-Dimethyl-1-pyrroline N-oxide (DMPO) and 2,2,6,6-tetramethylpiperidine (TEMP) were used as spin traps to distinguish ·OH and ·O_2_^−^ (in water or methanol) [43] and ^1^O_2_ (in water) [44], respectively (Figure 4). Characteristic signals corresponding to DMPO-·OH, DMPO-·O_2_^−^ and TEMP-^1^O_2_ adducts were all observed in the EPR spectra of the SiW_9_Mn_3_^II^/O_3_ system. These results indicate that ·OH, ·O_2_^−^ and ^1^O_2_ were generated during ozonation, contributing to the rapid oxidation of glucose at lower temperatures.

**Figure 5:**
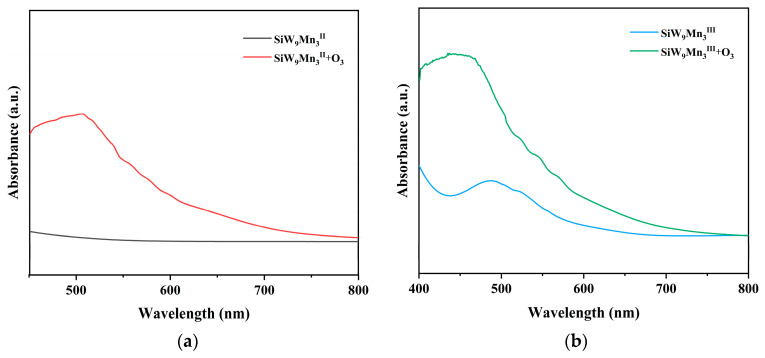
UV-Vis spectra of SiW_9_Mn_3_^II^ (**a**)/SiW_9_Mn_3_^III^ (**b**) in the presence of O_3_. Reaction conditions: 10 mg of catalyst, 10 mL of water, O_3_ flow rate of 30 mL/min for 1 h at room temperature.

Meanwhile, to determine interaction between SiW_9_Mn_3_^II^/SiW_9_Mn_3_^III^ with O_3_ or glucose, the UV-Vis spectra were obtained (Figure 5). It was observed that a new peak at approximately 500 nm, attributed to [SiW_9_Mn_3_^III^-·O_3_], appeared in the SiW_9_Mn_3_^II^/O_3_ system, while a peak at ~450 nm, assigned to [SiW_9_Mn_3_^IV^-·O_3_] [35,36], appeared in the SiW_9_Mn_3_^III^/O_3_ system. The appearance of these peaks in the Mn-POMs/O_3_ system confirmed the initial interaction between the POMs and O_3_. Furthermore, the UV-Vis spectra of SiW_9_Mn_3_^II^/SiW_9_Mn_3_^III^ with glucose at room temperature showed no new peaks, indicating that SiW_9_Mn_3_^II^ and SiW_9_Mn_3_^III^ exhibited low activity in oxidizing glucose under these conditions (Figure S1). Consequently, the Mn-POMs likely activated O_3_ to generate ROS via Equations (1)–(7), thereby contributing to glucose oxidation.

The ozonation of glucose resulted in a high yield of formic acid (FA) (Figure 1). Among all Mn-POMs, catalyst SiW_9_Mn_3_^II^ exhibited the highest FA yield of 79.3%, which was consistent with the conversion trend. The reaction products were identified as gluconic acid, xylose, glycolic acid (GA) and FA (Figure S2), with FA being the predominant product after 3 h.

**Scheme 1:**
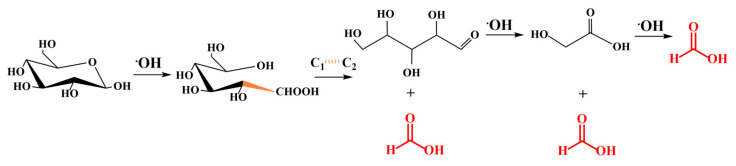
The possible pathway for glucose conversion over SiW_9_Mn_3_^II^/O_3_.

Based on the product distribution, it was proposed that glucose was initially oxidized by ROS to gluconic acid, followed by C1-C2 bond cleavage to yield xylose and FA. Subsequently, xylose was degraded to FA and glycolic acid (GA), with the final cleavage of GA to FA occurring in the presence of ·OH (Scheme 1).

Finally, the reaction conditions were optimized to 100 mg of glucose, 10 mg of SiW_9_Mn_3_^II^, 10 mL of H_2_O and an O_3_ flow rate of 30 min/min (Figure S3, Figure S4, Figure S5 and Figure S6). Catalyst SiW_9_Mn_3_^II^ was separated via decantation using methanol for subsequent reuse. The regenerated SiW_9_Mn_3_^II^ was collected by centrifugation with a recovery rate of 99.3%. Catalyst SiW_9_Mn_3_^II^ could be reused for at least six cycles; during this period, the glucose conversion decreased from 82.1% to 79.3%, and FA yields decreased from 76.5% to 71.6%. The total loss of SiW_9_Mn_3_^II^ relative to the fresh catalyst was approximately 6.0% (Figure S7). Moreover, IR, UV-Vis and XPS analyses of Mn-POMs before and after glucose oxidation revealed no significant changes, confirming the excellent stability of these catalysts under the reaction conditions (Figure S8, Figure S9 and Figure S10).

Furthermore, VOSO_4_ (0.0034 mmol), identified as the optimal homogeneous catalyst in Table S1, was selected for the ozonation of glucose. Under identical reaction conditions, VOSO_4_ achieved a glucose conversion of 75.4% with a formic acid (FA) yield of 65.8%. In comparison, catalyst SiW_9_Mn_3_^II^ exhibited superior performance in the ozonation of monosaccharides.

**Figure 6:**
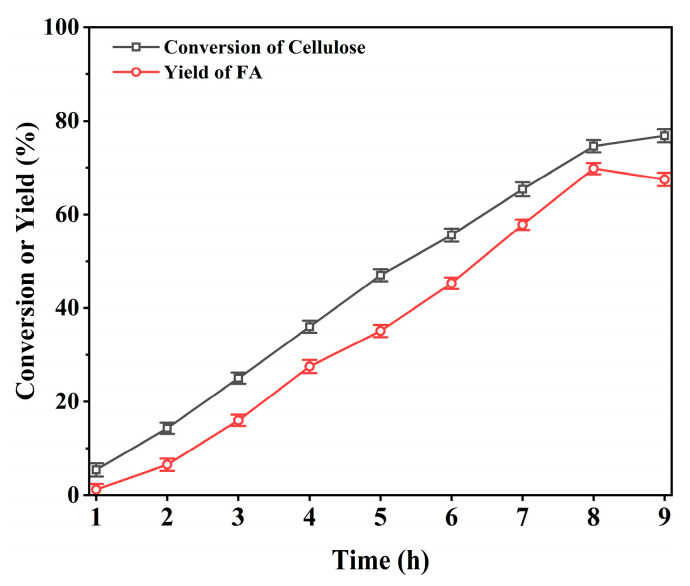
Catalytic activity of SiW_9_Mn_3_^II^ in ozonation of cellulose. Reaction conditions: 100 mg cellulose, 10 mg SiW_9_Mn_3_^II^, 10 mL water, O_3_ flow rate of 30 mL/min, 9 h, room temperature.

We further extended the application of the SiW_9_Mn_3_^II^/O_3_ system to cellulose conversion (Figure 6). A cellulose conversion of 75.6% was achieved at room temperature with a formic acid (FA) yield of 69.8%. This result was of significant value for the conversion of cellulosic polysaccharides into FA under mild reaction conditions.

## 3. Experimental Section

### 3.1. Chemicals and Measurements

All chemicals and solvents used in this work were of AR grade or better and used without further purification. The syntheses of K_x_[SiW_12−n_Mn_n_^m^O_(40−n)_] were carried out based on relevant reported procedures (see Supporting Information). The products were characterized by elementary analysis (Table S2), FTIR spectroscopy, TG/DTG, EPR [45,46] and mass spectrum (Figure S8, Figure S9, Figure S10, Figure S11, Figure S12, Figure S13, Figure S14 and Figure S15).

Elemental analyses were performed using a Leeman Plasma Spec (I) ICP-ES (Hudson, NH, USA) and a PerkinElmer 2400 CHN elemental analyzer (Shelton, CT, USA). IR spectra (4000–400 cm^−1^) were recorded as KBr pellets on an Agilent Cary 630 spectrometer (Santa Clara, CA, USA). X-ray photoelectron spectroscopy (XPS) was conducted on a Thermo ESCALAB 250X photoelectron spectrometer (Waltham, MA, USA) using an Al Kα source (1200 eV). EPR spectra were collected using a JES-FA 300 spectrometer (JEOL, Tokyo, Japan) operating at 9.05 GHz with a microwave power of 0.998 mW. UV-Vis diffuse reflectance spectra were recorded on a Cary 500 UV-Vis-NIR spectrophotometer. The concentrations of glucose and xylose (Xyl) were determined by HPLC (Shimadzu LC-16A, Kyoto, Japan) equipped with RID and UV detectors and an NH_2_ column. The mobile phase consisted of acetonitrile and water (volume ratio 4:1) at a flow rate of 1.0 mL/min. The column temperature was maintained at 25 °C, and the injection volume was 20 µL. Additionally, an ion chromatography system (ICS-930, Metrohm, Herisau, Switzerland) was employed to quantify the final oxidation products of glucose. The mobile phase was 0.5 mM H_2_SO_4_ at a flow rate of 0.5 mL/min, with an injection volume of 20 µL.

### 3.2. Oxidation of Glucose

#### 3.2.1. Oxidation of Glucose with O_3_

Glucose (100 mg), distilled water (10 mL) and the catalyst (10 mg) were added to a glass reactor equipped with an ozone bubbling device. The reaction was initiated by introducing O_3_ into the magnetically stirred mixture at a flow rate of 30 mL/min. After a reaction time of 3 h, the mixture was analyzed by HPLC and IC to quantify the concentration of glucose and FA. The catalyst was subsequently recovered using methanol.

#### 3.2.2. Quenching Experiments

Radical species generated during the oxidation process were identified under optimal conditions. Initially, different scavengers (i-PrOH [47], NaN_3_ [48] and BQ [44]) were added to 10 mL of water containing 100 mg of glucose and 10 mg of catalyst at a scavenger-to-catalyst molar ratio of 1:10. Subsequently, ozone was introduced at a flow rate of 30 mL/min for 3 h at room temperature. The role of reactive oxygen species (ROS) was evaluated by determining the conversion and yield of glucose.

#### 3.2.3. Oxidation of Cellulose with O_3_

The oxidation of cellulose was performed by mixing 100 mg of cellulose with 10 mg of catalyst in 10 mL of H_2_O. The mixture was stirred at 500 rpm for 9 h at room temperature with an O_3_ flow rate of 30 mL/min.

## 4. Conclusions

Mn-containing polyoxometalates (SiW_12−n_Mn_n_^m^, n = 1–3, m = II–IV) with different valence states were found to be active in activating O_3_ to generate reactive oxygen species (ROSs), demonstrating efficient ozonation activity in glucose oxidation. The catalytic activity was influenced by both the Mn content and its valence state, and a higher Mn loading and the presence of low-valent Mn(II) played pivotal roles. The initial interaction between SiW_9_Mn_3_^III^ and O_3_ to form [SiW_11_Mn^III^-O_3_·] contributed to the generation of ROSs including ·OH, ·O_2_^−^ and ^1^O_2_, which were identified by UV-Vis, EPR spectroscopy and quenching experiments. A 79.3% yield of formic acid (FA) was achieved from glucose via this ROS-mediated process, while a 69.8% yield was obtained directly from cellulose at room temperature. This work provides new insights into the application of POMs/O_3_ systems in biomass conversion.

## Data Availability

The original contributions presented in this study are included in the article and Supplementary Materials. Further inquiries can be directed to the corresponding authors.

## Supplementary Materials

The following supporting information can be downloaded at https://www.mdpi.com/article/10.3390/molecules31030467/s1, Preparation of Compounds; Table S1: Catalytic oxidation of glucose to FA using different types of catalysts in presence of O_2_; Figure S1: UV-Vis spectra of SiW_9_Mn_3_^II^ and SiW_9_Mn_3_^III^ with glucose; Figure S2: Product distribution in ozonation of glucose; Figure S3: Influence of ozone concentration on ozonation of glucose; Figure S4: Influence of glucose amount on glucose ozonation; Figure S5: Influence of catalyst amount on ozonation of glucose; Figure S6: Influence of reaction time on glucose ozonation; Table S2: The elemental analysis of K_x_[SiW_12-n_Mn_n_^m^O_(40−n)_] (SiW_12−n_Mn_n_^m^, n = 1–3, m = II–IV); Table S3: The elemental analysis of K_10_SiW_9_Mn_3_^II^O_37_ (1st and 2nd syntheses), (NH_4_)_10_SiW_9_Mn_3_^II^O_37_ and (C_16_H_36_N)_10_SiW_9_Mn_3_^II^O_37_; Figure S7: Recyclability of K_10_SiW_9_Mn_3_^II^O_37_ in glucose ozonation; Figure S8: IR spectra of Mn-POMs before (black) and after (red) the ozonation reaction; Figure S9: IR spectra of K_10_SiW_9_Mn_3_^II^O_37_ (1st and 2nd syntheses), (NH_4_)_10_SiW_9_Mn_3_^II^O_37_ and (C_16_H_36_N)_10_SiW_9_Mn_3_^II^O_37_. Figure S10: UV-Vis spectra of Mn-POMs before (black) and after (red) the ozonation reaction; Figure S11: XPS of SiW_9_Mn_3_^II^ (a) and SiW_9_Mn_3_^III^ (b) before and after the reaction, and results of two repeated syntheses (c); Figure S12: XRD spectra of K_10_SiW_9_Mn_3_^II^O_37_ (1st and 2nd syntheses), (NH_4_)_10_SiW_9_Mn_3_^II^O_37_ and (C_16_H_36_N)_10_SiW_9_Mn_3_^II^O_37_; Figure S13: TG/DTG curves of K_10_SiW_9_Mn_3_^II^O_37_·8H_2_O; Figure S14: EPR spectra of K_10_SiW_9_Mn_3_^II^O_37_ and (C_16_H_36_N)_10_SiW_9_Mn_3_^II^O_37_; Figure S15: ESI-MS of K_10_SiW_9_Mn_3_^II^O_37_.
