# Supplementary material for: Ozone-Oxidation of Glucose to Formic Acid over Polyoxmetalates
Source: Molecules. 2026 Jan 29;31(3):467. doi: 10.3390/molecules31030467 (PMC12899537; doi:10.3390/molecules31030467)
Supplement: Supplementary file 1 [file molecules-31-00467-s001.zip › molecules-4005688-supplementary.pdf]

Ozone-Oxidation of Glucose to Formic Acid over Polyoxmetalates

Xia Yu [1], Qiwen Wang [2,*], Haiyan Li [1], Tong Liu [1], Mengxue Xiu [1], Baiji Xue [1] and Linghe He [1,*]

1 Pharmaceutical Research and Development Center, Baicheng Medical College, Baicheng 137000, China

2 College of Resources and Environment, Jilin Agricultural University, Changchun 130118, China

* Corresponding author: Qiwen Wang; Fax: (+86)431-84533058; Tel: (86)431-84533058;

wangqw@jlau.edu.cn (Q.W.); hlh@bcmc.edu.cn (L.H.)

Preparation of Compounds

The synthesis of Mn-POMs based on the following equations:

$$K_4SiW_{12}O_{40} + OH^- + KCl \rightarrow K_8SiW_{11}O_{39} \quad (S1)$$

$$K_8SiW_{11}O_{39} + OH^- + KCl \rightarrow K_8SiW_{10}O_{36} \quad (S2)$$

$$K_4SiW_{12}O_{40} + OH^- + KCl \rightarrow K_9HSiW_9O_{34} \quad (S3)$$

$$K_8SiW_{11}O_{39} + Mn^{2+} \rightarrow K_6SiW_{11}MnO_{39} (H_2O) \quad (S4)$$

$$K_8SiW_{10}O_{36} + 2Mn^{2+} \rightarrow K_4SiW_{10}Mn_2O_{36} (H_2O)_2 \quad (S5)$$

$$K_{10}SiW_9O_{34} + 3Mn^{2+} \rightarrow K_{10}SiW_9Mn_3O_{37} (H_2O)_3 \quad (S6)$$

Preparation of α-$K_4SiW_{12}O_{40}$ (SiW_{12})

Sodium metasilicate (11g, 50mmol) is dissolved with magnetic stirring at room temperature in 100mL of distilled water (Solution A) Ref [31]. Sodium tungstate (182g, 0.55mol) is dissolved in 300mL of boiling distilled water (Solution B). To the boiling Solution B, a solution of 4 M HCl (l65 mL) is added drop by drop over 5min with vigorous stirring. Then solution A is added, followed quickly by addition of 50mL of 4 M HCI. The pH is 5 to 6. The solution is kept at 100 °C for 1 h. A solution of 1 M sodium tungstate (50mL, 50mmol) and, immediately thereafter, 80 mL of 4 M HCI are added. After cooling to room temperature, the solution is filtered. The pH is adjusted to 2 with aqueous 1 M KOH and 50g of solid KCl is added. The potassium salt is removed by filtering and dried in air. Yield: l30 g (75.0%).

Preparation of α-$K_7HSiW_{11}O_{39}$ (SiW_{11})

This compound was prepared by using the potassium salt of 12-tungstosilicate, obtained as Ref [32]. Potassium hydroxide (1.0 M) was slowly added to $K_4SiW_{12}O_{40}$ (0.1 M) until all the latter had been hydrolysed to $[SiW_{11}O_{39}]^{8-}$. The salt $K_7H[SiW_{11}O_{39}]$

was precipitated by the addition of saturated potassium chloride. The solid was filtered off and dried in air. Yield: 6 g (37.0%).

Preparation of α-$K_8SiW_{10}O_{36}$ (SiW_{10})

This compound was prepared by using the potassium salt of 11-tungstosilicate, obtained as Ref [33]. The pH of a solution prepared by dissolution of 15 g of $K_8SiW_{11}O_{39}$ in 150 mL of distilled water maintained at 25 °C was adjusted to 9.1 by addition of an aqueous solution of 2 mo1·L^{-1} K_2CO_3. The potassium salt of the 10-tungstosilicate was precipitated by addition of 20g of solid potassium chloride. The solid was filtered off and dried in air. Yield: 5g (70%).

Preparation of γ-$K_9HSiW_9O_{34}$ (SiW_9)

This compound was prepared by using the potassium salt of 12-tungstosilicate, obtained as Ref [32]. Potassium hydroxide (1.0 M) was slowly added to $K_4SiW_{12}O_{40}$ (0.1 M) until all the latter had been hydrolysed to $[SiW_9O_{34}]^{10-}$. The salt $K_9H[SiW_9O_{34}]$ was precipitated by the addition of saturated KCl. The solid was filtered off and dried in air. Yield: 5 g (35.0%).

Preparation of $K_x[SiW_{12-n}Mn_n^mO_{(40-n)}]$ ($SiW_{12-n}Mn_n^m$, $n = 1\text{-}3$, $m = II\text{-}IV$)

$K_{10}SiW_9Mn_3^{II}O_{37}$ ($SiW_9Mn_3^{II}$) Ref [34] To a solution of $Mn(CH_3COO)_2·4H_2O$ (3.3 g, 13.5 mmol) in 0.5 mol dm^{-3} sodium acetate (pH 6.5, 150 cm^3) was added SiW_9 (12.8 g, 4.5 mmol) as above. An initially formed yellow precipitate redissolved to give a yellow solution. After cation-exchange treatment, a solution of KCl (4 g) in water (12 cm^3) was added, producing a yellow precipitate which was filtered off and recrystallized from hot water (80 cm^3). Yield: 7 g (35.0%).

$K_7SiW_9Mn_3^{III}O_{37}$ ($SiW_9Mn_3^{III}$) Ref [35] A solution of SiW_9 (0.59 g, 0.2 mmol) in 5 mL of deionized water was quickly adjusted to pH = 3.9 with concentrated nitric acid. The pH of this solution was kept between 3.8 and 4.5 with nitric acid while solutions A and B as described below were added. The addition of solution A [0.1 g, 0.5 mmol, of manganese(II) acetate, $Mn(OAc)_2·2H_2O$, in 4 mL of water] afforded an orange solution. After the solution had been stirred for 2 min, solution B [potassium permanganate (0.02 g, 550 mmol) in 4 mL of water] was added dropwise. After about 30 min, the precipitate obtained by filtration was washed with isopropanol. Yield: 0.2 g (25.1%).

$K_8SiW_{10}Mn_2^{II}O_{38}$ ($SiW_{10}Mn_2^{II}$) Ref [34] A solution of $Mn(OAc)_2·4H_2O$ (1.6 g, 0.67 mmol) in water (15 mL) was added dropwise to a solution of SiW_{10} (10 g) in water (40 mL) at pH = 3-4 adjusted by acetic acid. After the solution had been stirred for 5 min, addition of solid potassium chloride (20 g) afforded about 10 g of orange precipitate upon cooling in an ice bath. The precipitate was dissolved in 15 mL of hot water and

reprecipitated in an ice bath. Yield: 10.0 g (34.9%).

$K_6SiW_{10}Mn_2^{III}O_{38}$ ($SiW_{10}Mn_2^{III}$) Ref [36] 13.8 g of $Mn(CH_3COO)_2{\cdot}4H_2O$ (56.3 mmol) were dissolved in 600 ml of acetate buffer (0.5 M, pH 6.5) at room temperature, this solution heated to 70 °C and 48.0 g (17.6 mmol) of solid SiW_9 were added, followed by 1 h stirring at the same temperature. KCl (13.0 g) was added. After one night, a thin brown residue was removed and the volume of the solution was reduced to 200 ml. The solution was filtered again and left in air. Substantial amount of a green-dark solid was formed and collected after three days. Yield: 31g (50.0%).

$K_6SiW_{11}Mn^{II}O_{39}$ ($SiW_{11}Mn^{II}$) Ref [37] 5.7 g (1.9 mmol) of SiW_{11} was dissolved in 30 mL of distilled water, and the resulting solution was heated to 95 °C. A solution of 0.6 g (2.2 mmol) of $Mn(CH_3COO)_2{\cdot}4H_2O$ was added dropwise with continuous stirring until a small amount of maroon precipitate appeared. The mixture was then cooled to room temperature and filtered to remove the precipitate. The filtrate was evaporated to a final volume of approximately 10 mL, followed by cooling to room temperature and addition of 30 mL of acetone. After the formation of a new precipitate, the mixture was subjected to filtration again. The collected filtrate was dried in an oven at 50 °C to yield $SiW_{11}Mn^{II}$. Yield: 3 g (47.0%).

$K_5SiW_{11}Mn^{III}O_{39}$ ($SiW_{11}Mn^{III}$) Ref [37] 11.5 g (3.8 mmol) of SiW_{11} was dissolved in 25 mL of distilled water, and 0.8 g (2.7 mmol) of $Mn(CH_3COO)_2{\cdot}4H_2O$ was dissolved separately in 5 mL of distilled water. The two solutions described above were mixed via dropwise addition, and the pH of the resulting mixture was adjusted to 6 using $KHCO_3$. The mixture was then heated in a water bath at 80 °C for 10 min, followed by the addition of 10 mL of NaClO solution. The solution was concentrated at room temperature and the resulting precipitate was collected and recrystallized in warm water at 60 °C to afford $SiW_{11}Mn^{III}$. Yield: 3 g (25.0%).

$K_4SiW_{11}Mn^{IV}O_{39}$ ($SiW_{11}Mn^{IV}$) Ref [36] 1.6 g (0.6 mmol) of $SiW_{11}Mn^{III}$ was dissolved in 20 mL of distilled water and stirred until a clear solution was formed. Subsequently, 2 g (7.4 mmol) of $K_2S_2O_8$ was added to the above solution. The mixture was heated to boiling and refluxed for 2 h, then cooled to room temperature and filtered to yield $SiW_{11}Mn^{IV}$. Yield: 1g (33.0%).

Table S1 Catalytic oxidation of glucose to FA using different types of catalysts in presence of O_2

	Catalysts	Sub.	Solvent	Reaction Conditions	Con. %	Yield %	Ref.
homogeneous catalyst	$VOSO_4$ 5.1mg	Glu 180mg	H_2O 20mL	140℃, 1h, 2MPa O_2	>99.0	53.0	[15]
	$NaVO_3$ 22mg	Glu 100mg	H_2O 5mL	160℃, 0.02h, 3MPa O_2	96.8	42.8	[16]
	$NaHPO_4$ 25mg	Glu 250mg	H_2O 50mL	100℃, 2h, 2MPa O_2	52.0	34.0	[12]
noble metal catalyst	RuO_2/Ti	Glu 18mg	NaOH 0.2M	80A/m^2, 0.25A	65.0	27.3	[17]
	$Ru(OH)_4$/r-Go 0.36mg	Glu 360mg	$FeCl_3$ 40mM	160℃, 1h, 0.5MPa O_2	95.6	50.0	[18]
metal oxide catalyst	MnO_x-100 50mg	Glu 100mg	H_2O 5mL	150℃, 2.5h, 3MPa O_2	100.0	81.1	[19]
	V_2O_5 25mg	Glu 250mg	H_2O 50mL	150℃, 3h, 2MPa O_2	100.0	30.0	[12]
	Mo-MnO_x 50mg	Glu 100mg	H_2O 5mL	160℃, 1.5h, 0.3MPa O_2	69.0	54.0	[20]
	CuCTAB/MgO 60mg	Glu 90mg	H_2O 5mL	120℃, 12 h, H_2O_2	100.0	65.0	[21]
	MgO 160mg	Glu 900mg	1-Hexanol 100mL	50℃, 4h, H_2O_2	90.5	78.6	[22]
metal-loaded catalysis	Mg-Al(3:1) 100mg	Glu 200mg	EtOH 20mL	70℃, 6h, H_2O_2	38.7	37.0	[23]
POMs catalysis	HPA-1 170mg	Glu 200mg	H_2O 20mL	180℃, 3h, 2MPa O_2	100.0	54.5	[24]
	HPA-2	Glu	H_2O	80℃, 26h,	>98.0	47.0	[9]

70mg	120mg	4mL	3MPa O_2			
HPA-3 136mg	Glu 500mg	H_2O 100mL	90°C, 8h, 3MPa O_2	97.0	56.0	[25]
HPA-4 136mg	Glu 500mg	H_2O 100mL	90°C, 8h, 3MPa O_2	94.0	52.0	[25]
HPA-5 126mg	Glu 5g	H_2O 100mL	90°C, 8h, 3MPa O_2	94.0	57.0	[25]
$[MIMPS]_3$-$HPVMo_{11}O_{40}$ 90mg	Glu 360mg	H_2O 5mL	180°C, 1h, 1MPa O_2	100.0	54.7	[26]

HPA-1: $H_4PMo_{11}VO_{40}\cdot32.5H_2O$; HPA-2: $H_5PV_2Mo_{10}O_{40}\cdot35H_2O$; HPA-3: $H_6PV_3Mo_9O_{40}\cdot10H_2O$; HPA-4: $H_7PV_4Mo_8O_{40}\cdot11H_2O$; HPA-5: $H_8PV_5Mo_7O_{40}\cdot12H_2O$.

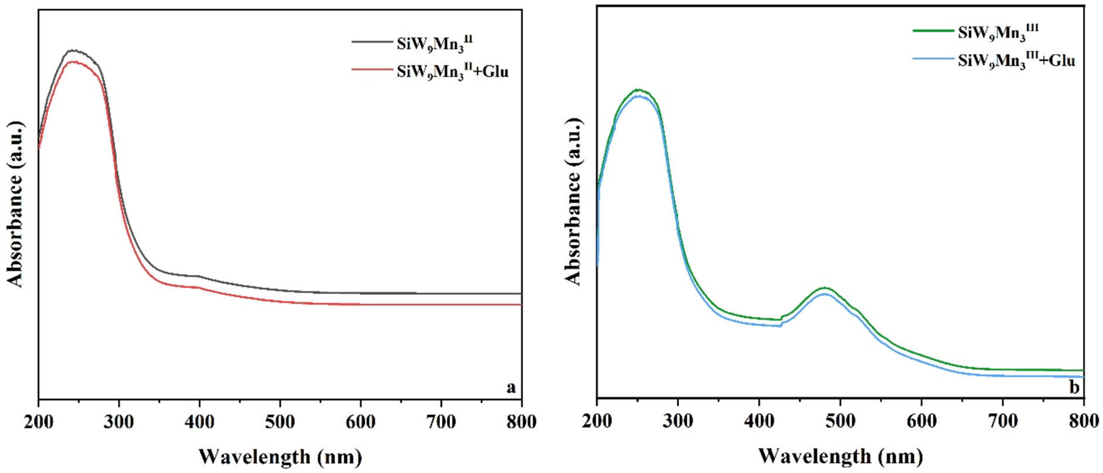

Figure S1. UV-Vis spectra of $K_{10}SiW_9Mn_3^{II}O_{37}$ and $K_7SiW_9Mn_3^{III}O_{37}$ with glucose. Reaction conditions: 100 mg glucose, 10 mg catalyst, 10 mL of H_2O, 1 h at room temperature.

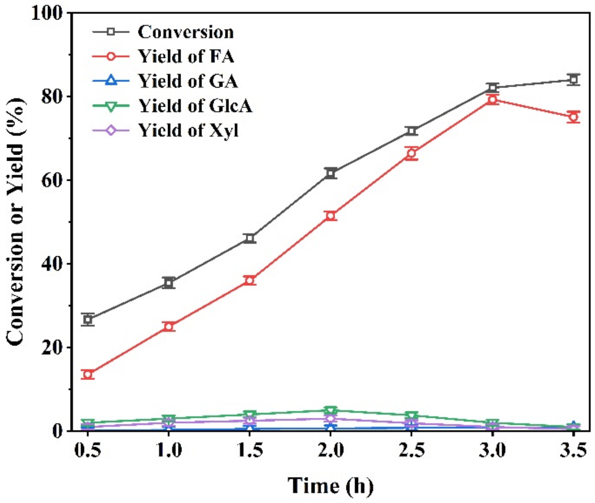

Figure S2. Product distribution in the ozonation of glucose. Reaction conditions: 100 mg glucose, 10 mg catalyst, 10 mL H_2O, O_3 flow rate of 30 mL/min for 3.5 h at room temperature.

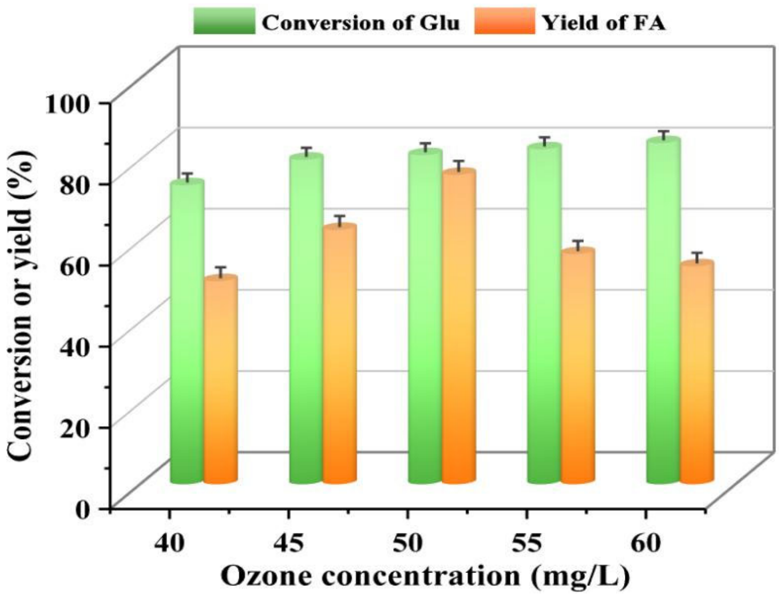

Figure S3. Influence of ozone concentration on ozonation of glucose. Reaction conditions: 100 mg glucose, 10 mg $K_{10}SiW_9Mn_3^{II}O_{37}$, 10 mL H_2O, O_3 flow rate of 30 mL/min for 3 h at room temperature.

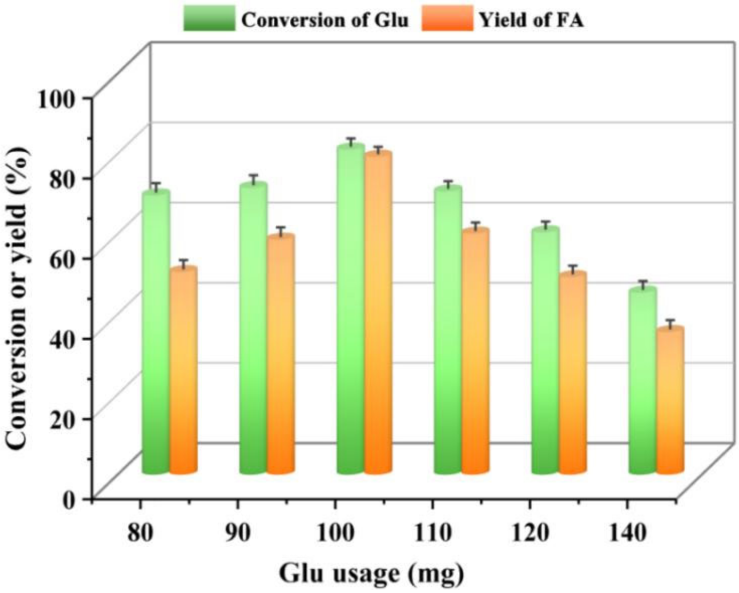

Figure S4. Influence of glucose amount on glucose ozonation. Reaction conditions: 10 mg $K_{10}SiW_9Mn_3^{II}O_{37}$, 10 mL H_2O, O_3 flow rate of 30 mL/min for 3 h at room temperature.

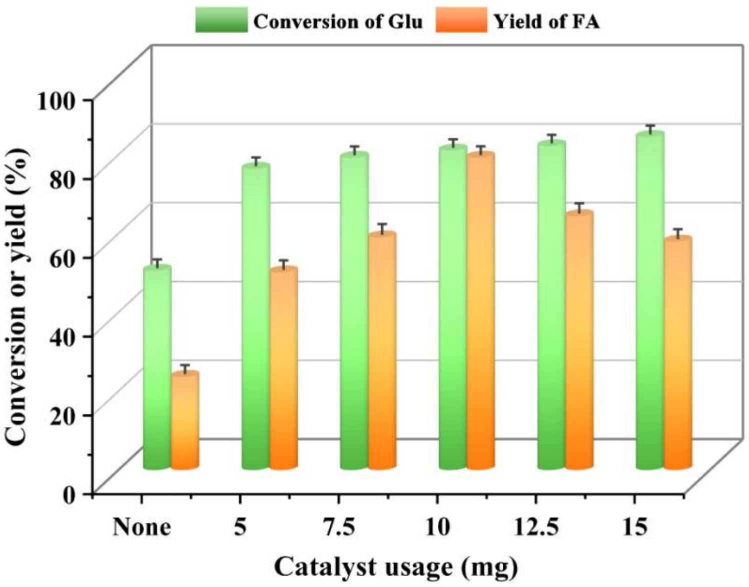

Figure S5. Influence of catalyst amount on ozonation of glucose. Reaction conditions: 100 mg glucose, 10 mL H_2O, O_3 flow rate of 30 mL/min for 3 h at room temperature.

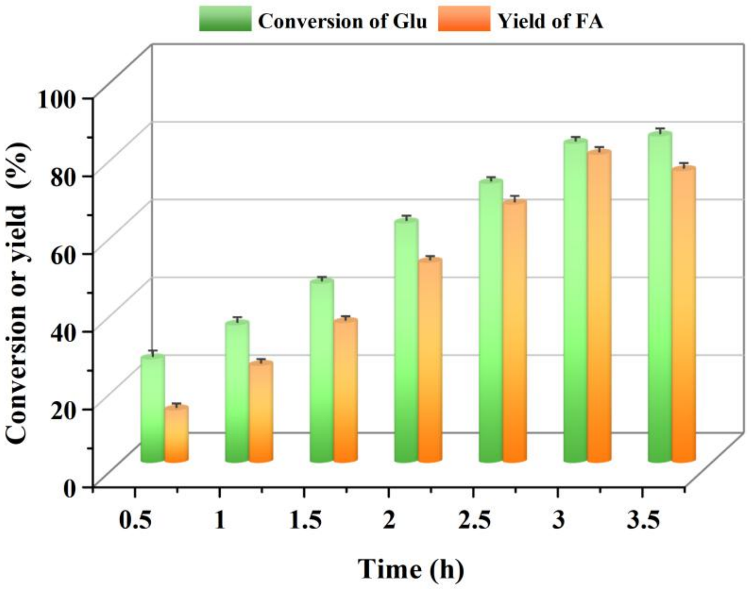

Figure S6. Influence of reaction time on glucose ozonation. Reaction conditions: 100 mg glucose, 10 mg $K_{10}SiW_9Mn_3^{II}O_{37}$, 10 mL H_2O, O_3 flow rate of 30 mL/min for 3 h at room temperature.

Table S2 The elemental analysis of $K_x[SiW_{12-n}Mn_n^mO_{(40-n)}]$ ($SiW_{12-n}Mn_n^m$, n = 1-3, m = II-IV)

Catalysts	Elementary results (calculated values in parenthesis)/%			
	K	Si	W	Mn
$K_{10}SiW_9Mn_3^{II}O_{37}$	13.92 (13.91)	1.03 (1.00)	58.47 (58.45)	5.84 (5.82)
$K_8SiW_{10}Mn_2^{II}O_{38}$	10.81 (10.79)	1.01 (0.97)	63.48 (63.45)	3.82 (3.80)
$K_6SiW_{11}Mn^{II}O_{39}$	7.91 (7.92)	1.03 (0.95)	68.25 (68.23)	1.87 (1.85)
$K_7SiW_9Mn_3^{III}O_{37}$	10.01(10.00)	1.06 (1.04)	70.01 (60.98)	6.09 (6.07)
$K_6SiW_{10}Mn_2^{III}O_{38}$	8.33 (8.32)	1.08 (1.00)	65.22 (65.21)	3.93 (3.90)
$K_5SiW_{11}Mn^{III}O_{39}$	6.70 (6.69)	1.02 (0.96)	69.13 (69.14)	1.90 (1.88)
$K_4SiW_{11}Mn^{IV}O_{39}$	5.43 (5.42)	1.01 (0.97)	70.08 (70.07)	1.92 (1.90)

Table S3 The elemental analysis of $K_{10}SiW_9Mn_3^{II}O_{37}$ (1st and 2nd syntheses), $(NH_4)_{10}SiW_9Mn_3^{II}O_{37}$ and $(C_{16}H_{36}N)_{10}SiW_9Mn_3^{II}O_{37}$

Catalysts (POMs include 8 H_2O)	Elementary results (calculated values in parenthesis)/%				
	K	N	Si	W	Mn
$K_{10}SiW_9Mn_3^{II}O_{37}$ (first)	13.10 (13.14)	-	0.97 (0.94)	55.59 (55.62)	5.55 (5.54)
$K_{10}SiW_9Mn_3^{II}O_{37}$ (second)	13.12 (13.14)	-	0.96 (0.94)	55.58 (55.62)	5.53 (5.54)
$(NH_4)_{10}SiW_9Mn_3^{II}O_{37}$	-	5.04 (5.07)	1.01 (1.02)	59.84 (59.86)	5.99 (5.96)
$(C_{16}H_{36}N)_{10}SiW_9Mn_3^{II}O_{37}$	-	0.29 (0.29)	0.57 (0.58)	33.91 (33.89)	3.37 (3.38)

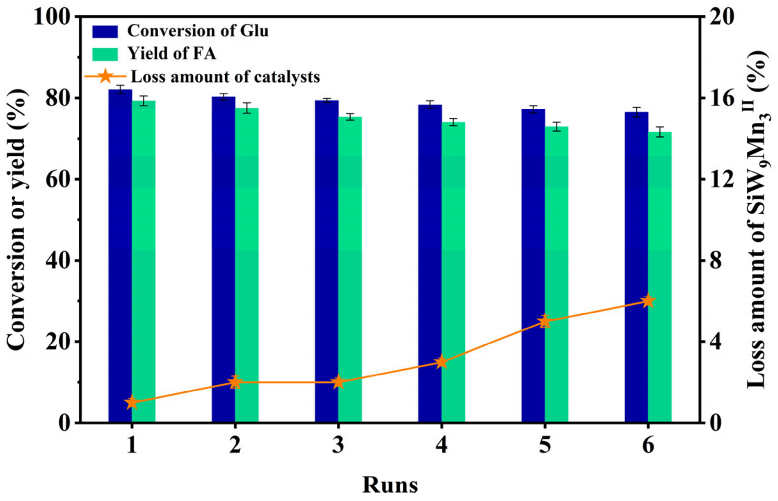

Figure S7. Recyclability of $K_{10}SiW_9Mn_3^{II}O_{37}$ in glucose ozonation. Reaction conditions: 100 mg glucose, 10 mg $K_{10}SiW_9Mn_3^{II}O_{37}$, 10 mL H_2O, O_3 flow rate of 30 mL/min for 3 h at room temperature.

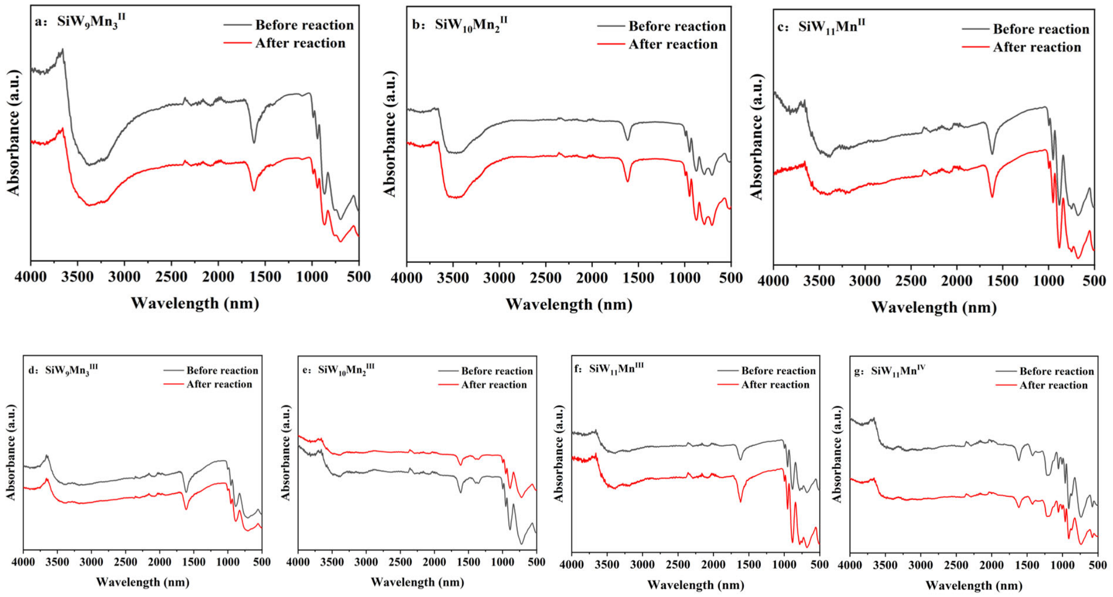

Figure S8. IR spectra of Mn-POMs before (black) and after (red) the ozonation reaction.

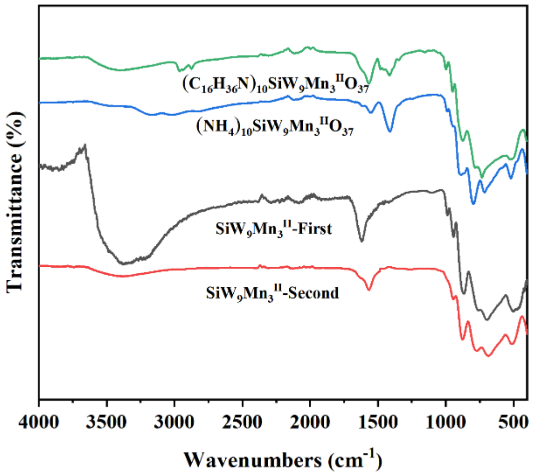

Figure S9. IR spectra of $K_{10}SiW_9Mn_3^{II}O_{37}$ (1st and 2nd syntheses), $(NH_4)_{10}SiW_9Mn_3^{II}O_{37}$ and $(C_{16}H_{36}N)_{10}SiW_9Mn_3^{II}O_{37}$.

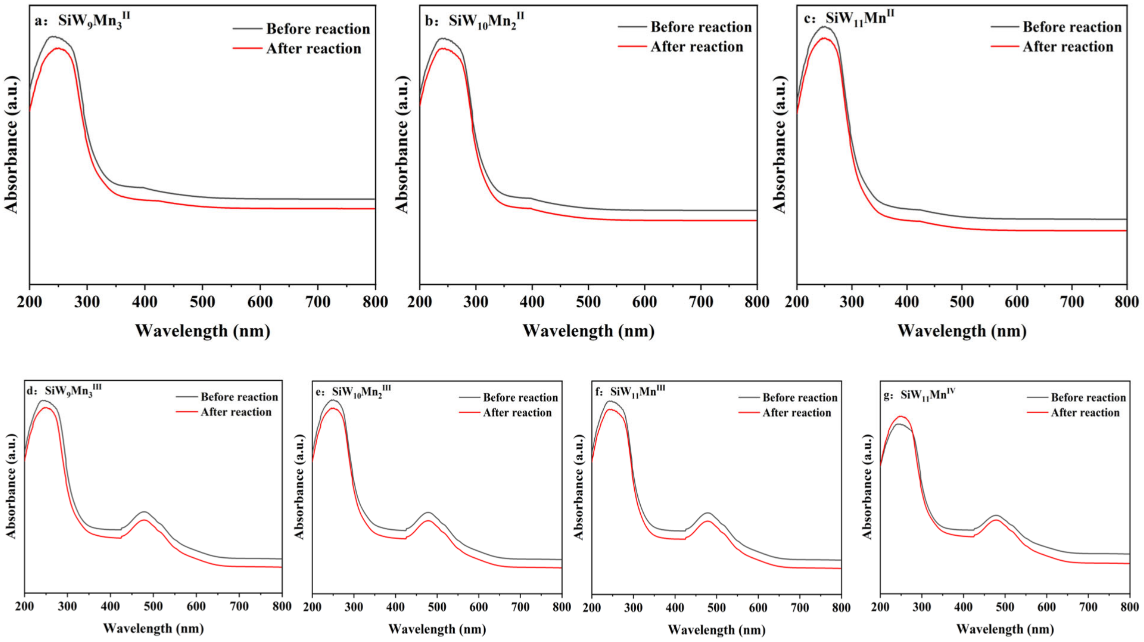

Figure S10. UV-Vis spectra of Mn-POMs before (black) and after (red) the ozonation reaction.

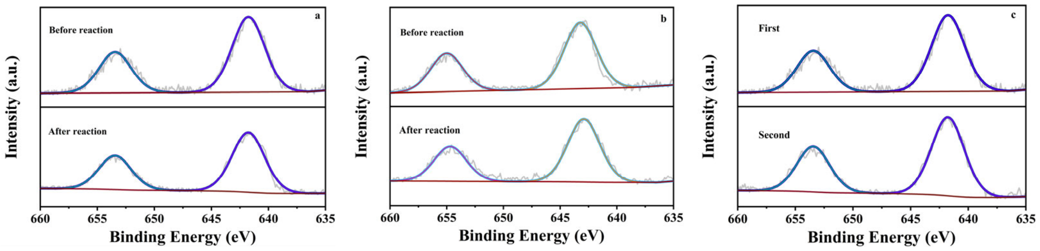

Figure S11. XPS of $K_{10}SiW_9Mn_3^{II}O_{37}$ (a) and $K_7SiW_9Mn_3^{III}O_{37}$ (b) before and after the reaction, and results of two repeated syntheses (c).

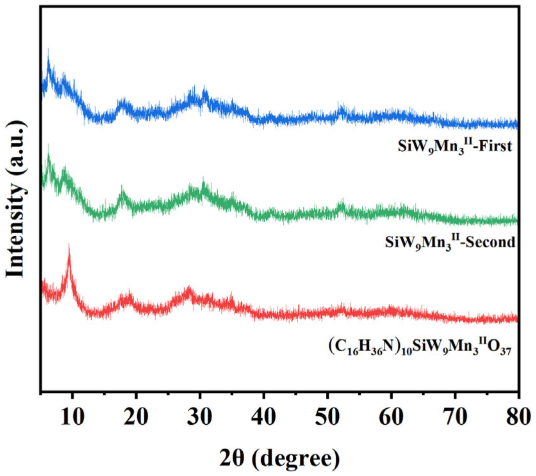

Figure S12. XRD spectra of $K_{10}SiW_9Mn_3^{II}O_{37}$ (1st and 2nd syntheses), $(NH_4)_{10}SiW_9Mn_3^{II}O_{37}$ and $(C_{16}H_{36}N)_{10}SiW_9Mn_3^{II}O_{37}$.

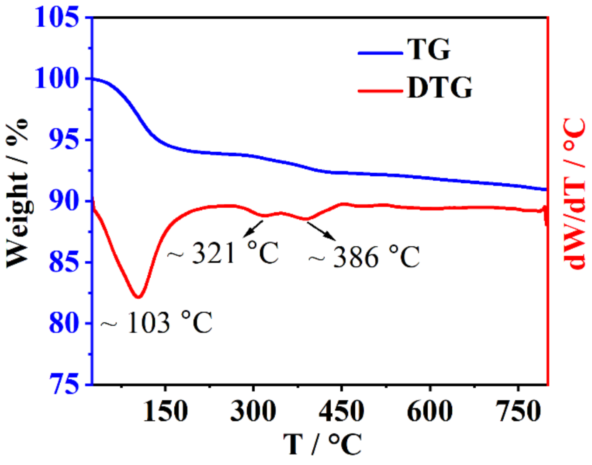

Figure S13. TG/DTG curves of $K_{10}SiW_9Mn_3^{II}O_{37}\cdot 8H_2O$.

From the TG/DTG curves, it could be seen that $SiW_9Mn_3^{II}$ was stable at room temperature, which lost about 4.7% of crystal water corresponding to the formula as $K_{10}SiW_9Mn_3O_{37}\cdot 8H_2O$, at 103 °C. And it decomposed at about 321~386 °C.

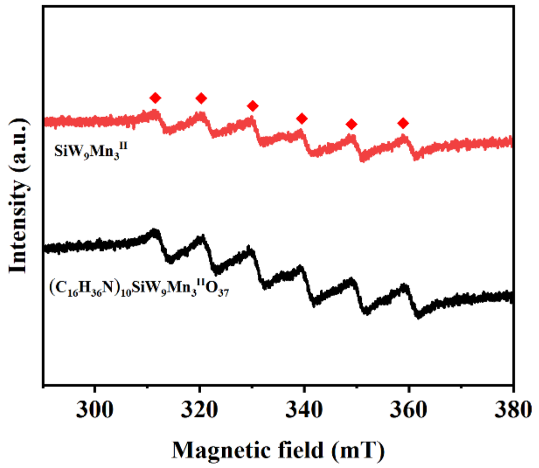

Figure S14. EPR spectra of $K_{10}SiW_9Mn_3^{II}O_{37}$ and $(C_{16}H_{36}N)_{10}SiW_9Mn_3^{II}O_{37}$.

Compared the EPR spectra of $(TBA)_{10}SiW_9Mn_3^{II}O_{37}$ and $K_{10}SiW_9Mn_3^{II}O_{37}$ [43], it displayed the same peaks for Mn^{2+} to conform the same patterns of Mn^{2+} in two compounds. This also determined the successful synthesis of $K_{10}SiW_9Mn_3O_{37}$ according to the Ref. [44]) for $(TBA)_{10}SiW_9Mn_3O_{37}$ synthesis.

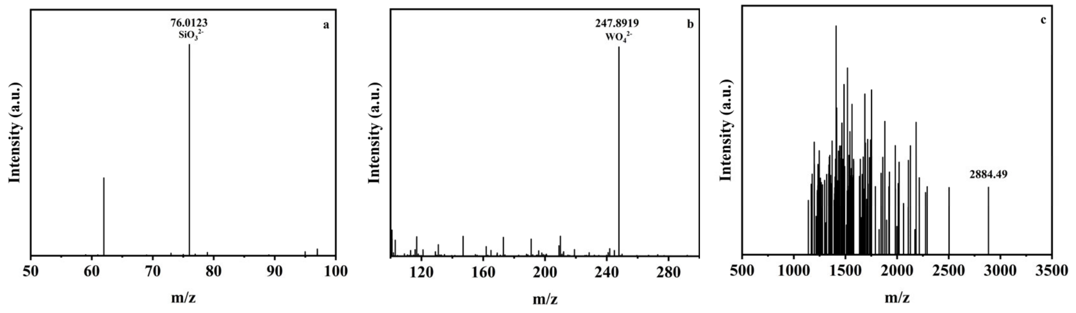

Figure S15. ESI-MS of Na_2SiO_3 (a), Na_2WO_4 (b), and $K_{10}SiW_9Mn_3^{II}O_{37}$ (c).

From ESI-MS, it could be seen 2884.49 (m/z) corresponding to the negative changed $K_{10}SiW_9Mn_3O_{37}$, determining the existence of $SiW_9Mn_3O_{37}^{10-}$ in the compound.
